# Renal functional and interstitial fibrotic assessment with non-Gaussian diffusion kurtosis imaging

**DOI:** 10.1186/s13244-022-01215-6

**Published:** 2022-04-08

**Authors:** Anqin Li, Guanjie Yuan, Yao Hu, Yaqi Shen, Xuemei Hu, Daoyu Hu, Zhen Li

**Affiliations:** Department of Radiology, Tongji Hospital, Tongji Medical College, Huazhong University of Science and Technology, 1095 Jiefang Avenue, Wuhan, 430030 Hubei China

**Keywords:** Magnetic resonance imaging, Diffusion kurtosis imaging, Renal dysfunction, Interstitial fibrosis

## Abstract

**Objectives:**

To evaluate the application value of diffusion kurtosis imaging (DKI) for monitoring renal function and interstitial fibrosis.

**Methods:**

Forty-two patients suspected of having primary nephropathy, hypertension or diabetes with impaired renal function were examined with DKI. DKI metrics of renal cortex and medulla on both sides of each patient were measured, including mean kurtosis (MK), axial kurtosis (Ka), radial kurtosis (Kr), mean diffusivity (MD) and fractional anisotropy (FA). The differences in DKI metrics between stable and impaired estimated glomerular filtration rate (eGFR) patients as well as between mild and severe interstitial fibrosis patients were compared. Correlations of DKI metrics with clinical indicators and pathology were analyzed. Diagnostic performance of DKI to assess the degree of renal dysfunction was analyzed.

**Results:**

Cortical MK, parenchymal Ka, MD and medullary FA were different in stable vs impaired eGFR patients and mild vs severe interstitial fibrosis patients (all *p* < .05). Negative correlation was found between Ka and eGFR (cortex: *r* = − 0.579; medulla: *r* = − 0.603), between MD and interstitial fibrosis (cortex: *r* = − 0.899; medulla: *r* = − 0.770), and positive correlation was found between MD and eGFR (cortex: *r* = 0.411; medulla: *r* = 0.344), between Ka and interstitial fibrosis (cortex: *r* = 0.871; medulla: *r* = 0.844) (all *p* < .05). DKI combined with mean arterial blood pressure (MAP) and urea showed good diagnostic power for assessing the degree of renal dysfunction (sensitivity: 90.5%; specificity: 89.5%).

**Conclusions:**

Noninvasive DKI has certain application value for monitoring renal function and interstitial fibrosis.

## Key points

- Non-Gaussian DKI is a non-invasive method to evaluate renal function and pathology.
- There are statistically significant differences in cortical MK, parenchymal Ka, MD and medullary FA between patients with stable and impaired eGFR and between patients with mild and severe interstitial fibrosis.
- Parenchymal Ka and MD are correlated with clinicopathological characteristics such as eGFR and interstitial fibrosis.
- DKI combined with MAP and urea might serve as more accurate markers for assessing the degrees of renal dysfunction.
- DKI has potential value in monitoring renal function and interstitial fibrosis.

## Introduction

Once the renal function of primary nephropathy further declines, it indicates disease progression and poor prognosis [1–3]. In addition, systemic diseases such as hypertension and diabetes can also cause kidney involvement due to target organ damage or complications [4, 5]. Studies have shown that estimated glomerular filtration rate (eGFR) and proteinuria are independent risk factors for all-cause mortality, cardiovascular disease mortality and end-stage renal disease [6–9]. Renal fibrosis is the optimal predictor of chronic kidney disease progression [10, 11]. Therefore, monitoring renal function and fibrosis and early recognition of renal dysfunction are essential to initiate appropriate treatment to prevent from serious outcomes. At present, surveillance and follow-up examinations of renal function are mainly based on serum creatinine (Scr) and urine protein. However, Scr is neither sensitive nor specific [12]. The collection of urine sample is often not standardized, which makes the test results biased [13]. Renal biopsy is currently the gold standard for assessing the severity of fibrosis, but it is invasive, prone to complications and susceptible to sampling bias [14]. Therefore, noninvasive MRI markers are preferable for timely identification of progressive renal dysfunction and assessment of renal fibrosis.

Non-Gaussian model diffusion kurtosis imaging (DKI) is an extension of diffusion tensor imaging (DTI). Only small changes in data acquisition and processing can better reflect the microstructure characteristics of the organization [15–17]. In this model, the deviation from the free diffusion of water molecules can be measured, and the kurtosis index including mean kurtosis (MK), axial kurtosis (Ka) and radial kurtosis (Kr) can be obtained [18]. Recently, several studies have confirmed that DKI can be used for noninvasive assessment of renal function and pathology [16, 19–24]. In particular, Mao et al. reported that DKI is feasible for evaluating the alterations of renal function and assessing the degree of renal pathological injury in CKD patients [23]. Liu et al. showed that ADC value from DKI model has predictive value for the progression of CKD, which could be a promising noninvasive technique in the follow-up of CKD patients [20]. However, there are still few related experiments and clinical studies, and the application value of DKI needs further verification.

We preliminarily determined that DKI has a certain correlation with renal function and fibrosis in an animal experiment, which indicated the applicability of DKI in measuring and monitoring longitudinal progression of kidney impairment [25]. The purpose of this study was to further investigate whether DKI can provide reliable diagnosis for progressive renal dysfunction and fibrosis in the clinic.

## Materials and methods

### Study population

**Fig. 1:**
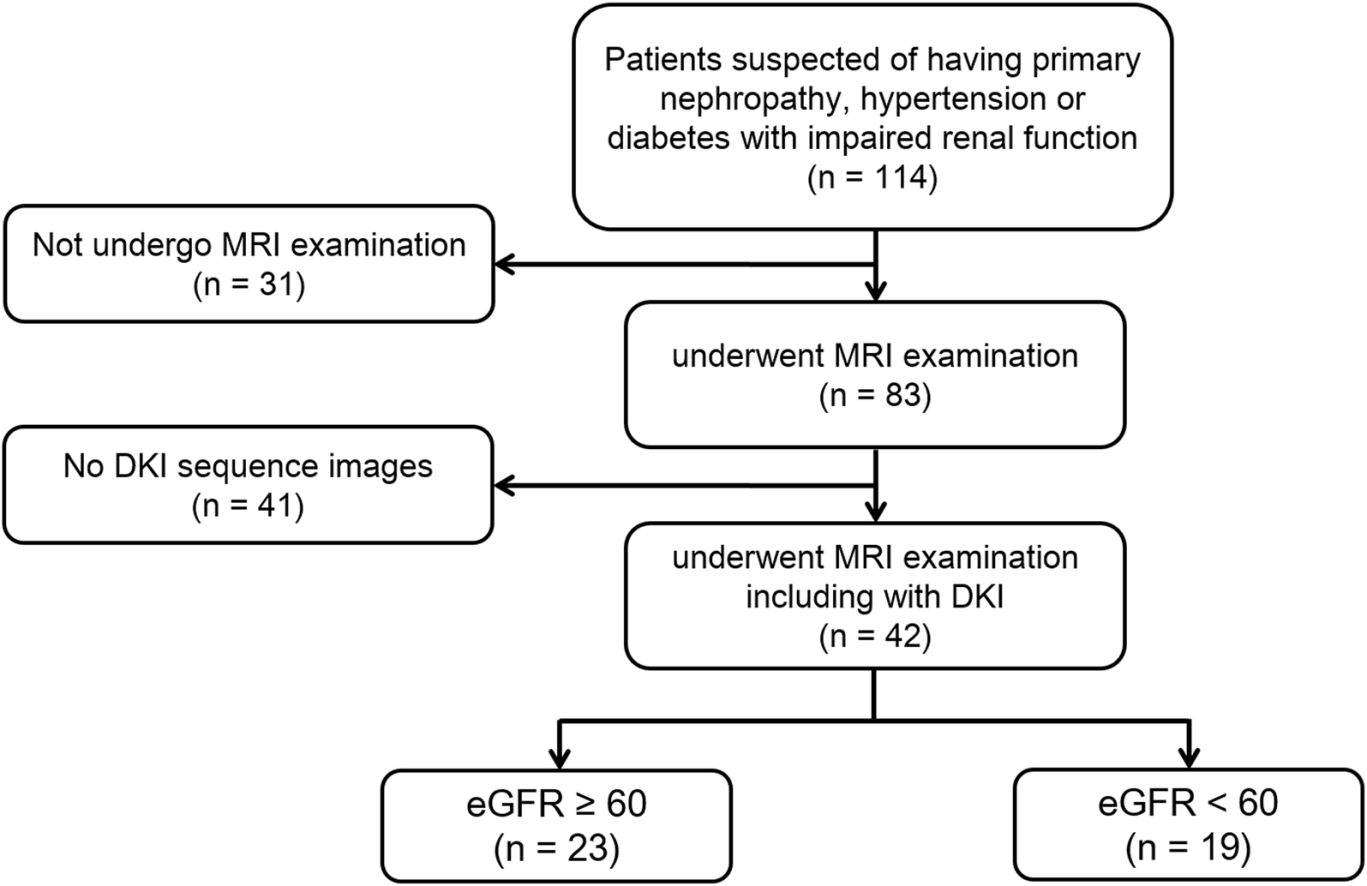
The flow chart of study population

This retrospective study was approved by our Institutional Review Board, and written informed consent was waived. The study included inpatients who sought medical advice in the Department of Nephrology, Cardiology and Endocrinology of our Hospital from October 2019 to January 2020. The clinical manifestations of patients were chronic renal insufficiency, nephrotic syndrome, nephritis syndrome, asymptomatic hematuria and/or proteinuria regardless of eGFR and hypertension or diabetes with impaired renal function (eGFR < 90 ml/min/1.73 m^2^). The flow chart of study population is presented in Fig. 1.

Patients’ demographics and the closest laboratory values within 3 days of MRI examination were obtained by reviewing electronic medical records, including gender, age, body mass index (BMI), mean arterial blood pressure (MAP), blood glucose, urea, serum creatinine, cystatin C, eGFR, 24-h urine volume, urinary total microprotein (mTP), urinary microalbumin (mALB), urine creatinine and urinary microalbumin/creatinine ratio (UALB/Cr). The eGFR was calculated according to the formula recommended by the Chronic Kidney Disease Epidemiology Collaboration (CKD-EPI): GFR = 141 × min(Scr/κ, 1)^α^ × max(Scr/κ, 1)^−1.209^ × 0.993^Age^ × 1.018 [if female] × 1.159 [if black], where Scr is serum creatinine, κ is 0.7 for females and 0.9 for males, α is -0.329 for females and −0.411 for males, min indicates the minimum of Scr/κ or 1, and max indicates the maximum of Scr/κ or 1 [26]. All subjects were divided into two groups according to the eGFR: group 1, patients with good or stable renal function (eGFR ≥ 60 ml/min/1.73 m^2^); group 2, patients with moderately or severely impaired renal function (eGFR < 60 ml/min/1.73 m^2^). If the patient underwent percutaneous renal biopsy under the guidance of B-ultrasound within 1 week of MRI examination, the pathological results were recorded. Interstitial fibrosis was evaluated by using a semi-quantitative assessment method (two-point scale): 0 (mild fibrosis, < 25%), 1 (moderate fibrosis, 25–50%) and 2 (severe fibrosis, > 50%).

### MRI protocol

MRI were performed with the patients on a 3T MRI scanner (Discovery 750, GE Healthcare, Milwaukee, USA) using a 32-channel torso array coil. There was no special preparation before the MRI examination, such as fasting or restricting fluid intake. For anatomical reference, prior routine scan of the kidney: (1) coronal SSFSE (single shot fast spin echo) T2-weighted image: repetition time/echo time, 1840/68 ms; field of view, 360 × 360 mm; matrix, 288 × 288; slice thickness, 5 mm; intersection gap, 1 mm; (2) axial LAVA-FLEX (liver imaging with volume acceleration-flexible) T1-weighted image: repetition time/echo time, 3.8/1.7 ms; field of view, 360 × 324 mm; matrix, 260 × 210; slice thickness, 4 mm; (3) axial PROPELLER FSE (propeller fast spin echo) T2-weighted image: repetition time/echo time, 8571/69 ms; field of view, 360 × 360 mm; matrix, 320 × 320; slice thickness, 5 mm; intersection gap, 1 mm.

DKI was performed in the axial plane using the SE-EPI (spin echo-echo planar imaging) diffusion sequence to acquire two images with *b* = 0 and images with b values of 1250 and 2500 s/mm^2^ along 25 diffusion encoding directions. Other scanning parameters: number of excitation, 2; repetition time, 3000 ms; echo time, minimum; slice thickness, 5 mm; intersection gap, 0 mm; field of view, 360 × 252 mm; matrix, 128 × 128; bandwidth, 250 kHz. The scan time of DKI sequence was 5 min and 15 s.

### Image analysis

The acquired DKI images were transferred to a workstation (Advantage Workstation, version 4.6, GE Healthcare) and processed using Functool software. The DKI metrics were calculated by the following formula [15, 17]:

$$\ln \left[ {S\left( {n,b} \right)/S_{0} } \right] = - b\mathop \sum \limits_{i = 1}^{3} \mathop \sum \limits_{j = 1}^{3} n_{i} n_{j} D_{ij} + \frac{1}{6}b^{2} \overline{D}^{2} \mathop \sum \limits_{i = 1}^{3} \mathop \sum \limits_{j = 1}^{3} \mathop \sum \limits_{k = 1}^{3} \mathop \sum \limits_{l = 1}^{3} n_{i} n_{j} n_{k} n_{l} W_{ijkl}$$

Here, $S\left( {n,b} \right)$ is the signal intensity for value *b* (*b* ≠ 0) along direction *n*, ${\text{S}}_{0}$ is the signal intensity for b = 0, $\overline{D}$ is the mean diffusivity, $n_{i} \left( {i = 1,2,3} \right)$ is the component of the diffusion direction vector *n*, $D_{ij}$ and $W_{ijkl}$ are the second-order diffusion tensor D and fourth-order kurtosis tensor *K*, respectively. Pseudocolor maps of DKI metrics are automatically generated, including mean kurtosis (MK), axial kurtosis (Ka), radial kurtosis (Kr), mean diffusivity (MD) and fractional anisotropy (FA).

Two independent blinded observers, with 8 and 23 years of experience in genitourinary radiology, respectively, reviewed all the MR images. Oval regions of interest (ROIs) were defined in three different slices of the upper pole, hilus and lower pole of each kidney (including cortex and medulla, respectively) on the *b* = 0 s/mm^2^ image and automatically copied to pseudocolor maps of MK, Ka, Kr, MD and FA. The DKI metrics values of each patient were obtained by calculating the average of three slices.

### Statistical analysis

Statistical analysis was performed with SPSS software (version 19.0, IBM). Kolmogorov–Smirnov test was used to test whether continuous variables follow a normal distribution. The variables of normal distribution were shown as mean ± standard deviation, and the variables of non-normal distribution were shown as median (range). Categorical variables were shown as percentages. The paired Student’s *t* test and independent Student’s *t* test or Mann–Whitney *U* test were used to compare the DKI metrics between the left and right kidney and between the cortex and medulla. The independent Student’s *t* test, Mann–Whitney *U* test or Chi-squared test was used to compare the sex ratio, age, BMI, MAP, blood glucose, urea, Scr, cystatin C, 24 h urine volume, mTP, mALB, urine creatinine, UALB/Cr, the degree of interstitial fibrosis between stable and impaired eGFR groups. DKI metrics was compared between stable and impaired eGFR groups as well as between mild and severe interstitial fibrosis groups. Correlation of DKI metrics with clinical indicators and pathology was assessed using Pearson correlation coefficient or Spearman rank correlation coefficient. Receiver operating characteristic (ROC) analysis was used to determining the diagnostic performance of DKI in assessing the degree of renal dysfunction. *p* < 0.05 was considered statistically significant.

## Results

### Demographic and clinicopathological characteristics of patients

A total of 42 patients underwent DKI, and 12 of them underwent renal biopsy. The mean values and standard deviation of eGFR were 90.9 ± 27.5 in patients with good or stable renal function group and 27.4 ± 13.8 in patients with moderately or severely impaired renal function group. MAP, urea, Scr, cystatin C, mTP, mALB, UALB/Cr and the degree of interstitial fibrosis in impaired eGFR group were significantly higher than those in stable eGFR group (*p* = 0.005, *p* < 0.001, *p* < 0.001, *p* = 0.017, *p* = 0.018, *p* = 0.009, *p* = 0.016, *p* = 0.001, respectively). There was no significant difference in terms of sex ratio, age, BMI, blood glucose, 24 h urine volume or urine creatinine between the two groups (all *p* > 0.05). The demographics, clinical and pathological characteristics of patients in the two groups are shown in Table 1.

**Table 1:** The demographics, clinical and pathological characteristics of patients in stable and impaired eGFR patients

	eGFR ≥ 60	eGFR < 60	*p*
	(*n* = 23)	(*n* = 19)	
eGFR (ml/min/1.73 m^2^)	90.9 ± 27.5	27.4 ± 13.8	< 0.001
Male: Female	13: 10	13: 6	0.429
Age (years)	51 ± 23	48 ± 19	0.720
BMI (kg/m^2^)	22.00 ± 4.56	22.48 ± 3.47	0.709
MAP (mmHg)	96 ± 23	116 ± 18	0.005
Blood glucose* (mmol/L)	5.53 (4.40–15.18)	5.42 (4.10–13.37)	0.487
Urea (mmol/L)	5.65 ± 1.87	12.89 ± 6.34	< 0.001
Serum creatinine (µmol/L)	79 ± 19	284 ± 164	< 0.001
Cystatin C (mL)	1.27 ± 0.44	4.06 ± 2.04	0.017
24 h urine volume (mL)	1836 ± 1288	1728 ± 524	0.811
24 h urinary mTP (mg/L)	498.5 ± 375.0	4760.5 ± 4653.7	0.018
24 h urinary mALB (mg/L)	286.1 ± 259.7	3104.9 ± 2705.0	0.009
Urine creatinine (µmol/L)	4839.56 ± 5296.71	5593.14 ± 5797.25	0.772
UALB/Cr (µg/mg)	331.31 ± 425.66	2569.15 ± 2711.16	0.016
Interstitial fibrosis^†^ (*n* = 12)			0.001
< 25%	7 (100%)	0 (0%)	
25–50%	0 (0%)	0 (0%)	
> 50%	0 (0%)	5 (100%)	

*eGFR* estimated glomerular filtration rate, *BMI* body mass index, *MAP* mean arterial blood pressure, *mTP* urinary total microprotein, *mALB* urinary microalbumin, *UALB/Cr* urinary microalbumin/creatinine. Unless otherwise stated, data are mean and standard deviation

^*^Data are median with interquartile range in parentheses

^†^Data are the numbers of patients, and numbers in parentheses are percentages

### DKI metrics of patients with different degrees of renal dysfunction and fibrosis

**Fig. 2:**
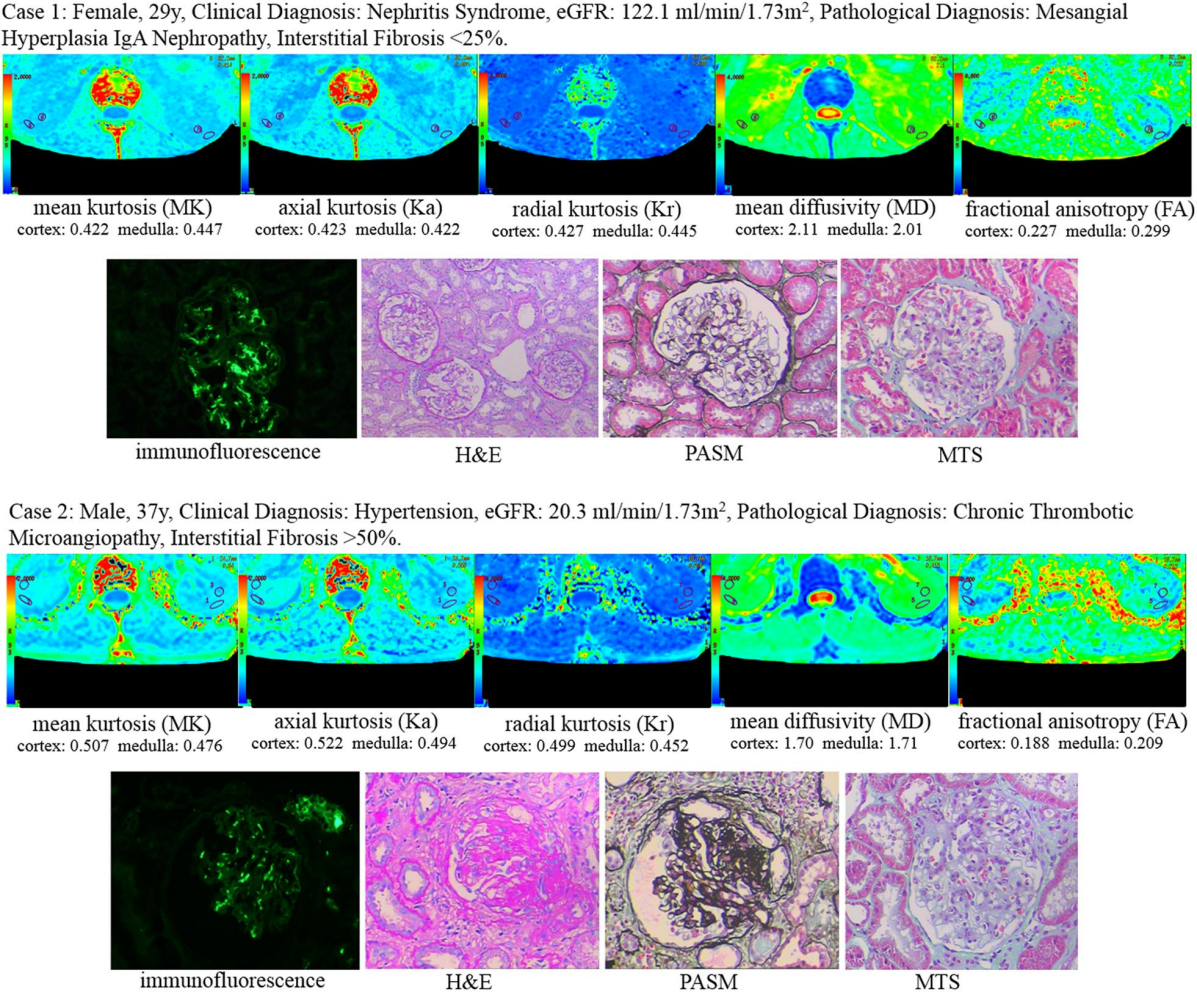
Example of DKI metrics pseudocolor maps and pathological images in a 29-year-old female nephritis syndrome patient with normal renal function (eGFR: 122.1 ml/min/1.73 m^2^) and a 37-year-old male hypertension patient with renal insufficiency (eGFR: 20.3 ml/min/1.73 m^2^). Increased cortical MK and parenchymal Ka and decreased parenchymal MD and medullary FA were determined in patient 2 compared with patient 1. The renal pathology (Immunofluorescence of IgA, Haematoxylin–Eosin, Periodic Acid–Silver Methenamine and Masson’s Trichrome stain; magnification, × 400) results indicated mild mesangial hyperplasia IgA nephropathy with glomerulosclerosis, tubular atrophy or interstitial fibrosis < 25% in patient 1 and chronic thrombotic microangiopathy with glomerulosclerosis, tubular atrophy and interstitial fibrosis > 50% in patient 2

Representative DKI metrics pseudocolor maps and pathological images of patients with different degrees of renal dysfunction and fibrosis are shown in Fig. 2.

Table 2 summarizes the DKI metrics of the cortex and medulla on the left and right kidneys. No significant difference was found on MK, Ka, Kr, MD or FA between the left and right kidneys (all *p* > 0.05). MK and Kr of renal cortex were significantly higher than those of renal medulla (*p* = 0.006, *p* = 0.024, *p* < 0.001, respectively), MD and FA of renal cortex were significantly lower than that of renal medulla (*p* < 0.001). However, there was no significant difference on Ka between cortex and medulla (*p* = 0.285). The interobserver agreements between the two observers were excellent (Table 3).

**Table 2:** The DKI metrics of the cortex and medulla on the left and right kidneys

	Cortex			Medulla			*p*
	Left kidney	Right kidney	*p*	Left kidney	Right kidney	*p*	
Mean kurtosis (MK)	0.474 ± 0.046	0.483 ± 0.052	0.124	0.465 ± 0.047	0.478 ± 0.044	0.335	0.006
Axial kurtosis (Ka)	0.477 ± 0.058	0.473 ± 0.045	0.584	0.474 ± 0.060	0.470 ± 0.050	0.612	0.285
Radial kurtosis (Kr)	0.448 ± 0.050	0.455 ± 0.072	0.078	0.434 ± 0.050	0.451 ± 0.055	0.561	0.024
Mean diffusivity (MD)	1.905 ± 0.259	1.829 ± 0.186	0.472	1.919 ± 0.275	1.842 ± 0.224	0.547	< 0.001
Fractional anisotropy (FA)	0.218 ± 0.045	0.263 ± 0.062	0.884	0.219 ± 0.046	0.265 ± 0.058	0.831	< 0.001

All metrics are dimensionless, except for MD (mm^2^/ms)

**Table 3:** The interobserver agreements between two radiologists of DKI metrics

	Intraclass correlation coefficient	95% Confidence intervals
**MK**		
Cortex	0.978	0.960–0.988
Medulla	0.980	0.962–0.989
**Ka**		
Cortex	0.987	0.976–0.993
Medulla	0.977	0.958–0.988
**Kr**		
Cortex	0.982	0.966–0.990
Medulla	0.988	0.978–0.994
**MD**		
Cortex	0.963	0.933–0.980
Medulla	0.961	0.929–0.979
**FA**		
Cortex	0.983	0.968–0.991
Medulla	0.989	0.979–0.994

**Fig. 3:**
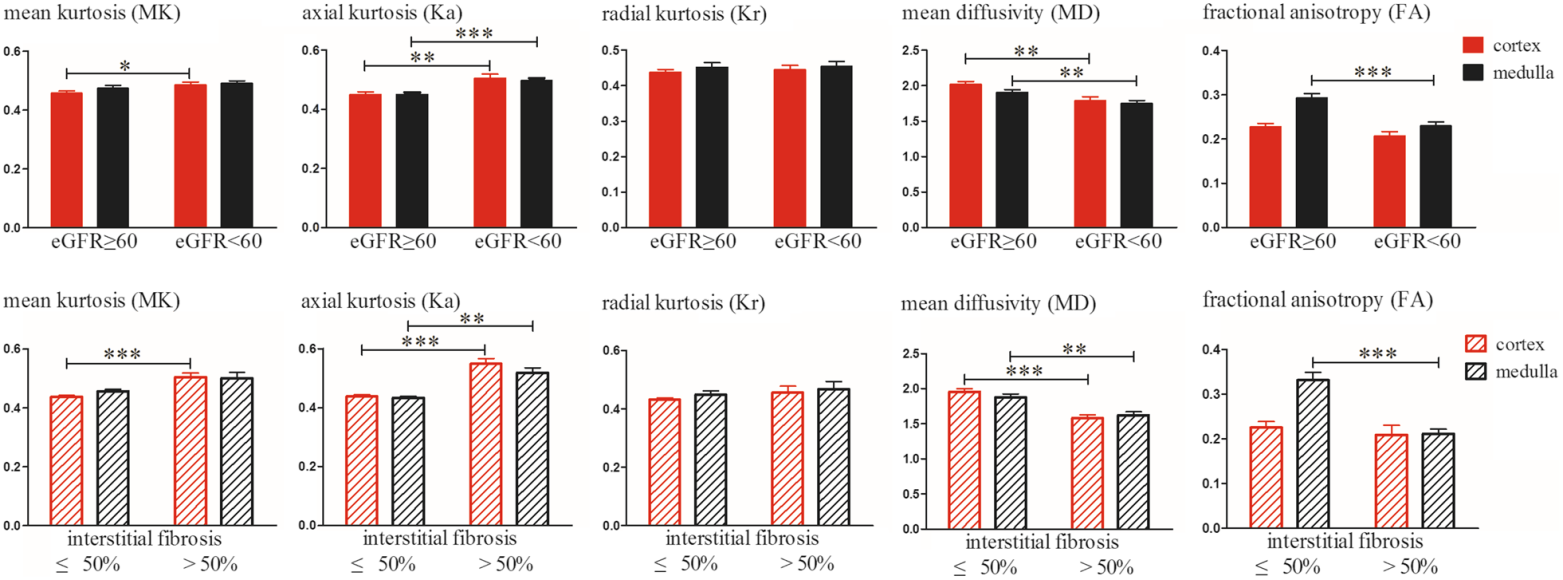
Diffusion kurtosis imaging (DKI) metric values in renal cortex and medulla between patients with different degrees of renal dysfunction and interstitial fibrosis. There were significant differences on cortical MK, parenchymal Ka, MD and medullary FA between the two groups. ^*^p < 0.05, ^**^p < 0.01, ^***^p < 0.001

The DKI metrics between patients with different degrees of renal dysfunction and fibrosis are shown in Fig. 3. MK and Ka of renal cortex in patients with impaired renal function were significantly higher than those in patients with stable renal function (*p* = 0.030, *p* = 0.001), MD of renal cortex in impaired eGFR groups was significantly lower than that in stable eGFR group (*p* = 0.003). However, there was no significant difference on Kr or FA of renal cortex between patients with different degrees of renal dysfunction (*p* = 0.642, *p* = 0.095). Compared with the patients with stable renal function, Ka of renal medulla in patients with impaired renal function was significantly higher (*p* = 0.001), MD and FA of renal medulla in patients with impaired renal function were significantly lower (*p* = 0.008, *p* < 0.001). However, there was no significant difference on MK or Kr of renal medulla between patients with different degrees of renal dysfunction (*p* = 0.236, *p* = 0.923). Cortical MK and parenchymal Ka in severe interstitial fibrosis patients were significantly higher than those in mild interstitial fibrosis patients (MK: *p* < 0.001; Ka: cortex: *p* < 0.001; medulla: *p* = 0.006), parenchymal MD and medullary FA in severe interstitial fibrosis patients were significantly lower than that in mild interstitial fibrosis patients (MD: cortex: *p* < 0.001; medulla: *p* = 0.006; FA:* P* < 0.001). However, there was no significant difference on medullary MK, parenchymal Kr or cortical FA between patients with different degrees of interstitial fibrosis (MK: *p* = 0.093; Kr: cortex: *p* = 0.329 medulla: *p* = 0.506; FA: *p* = 0.503).

### Correlation of DKI with clinicopathological characteristics

**Fig. 4:**
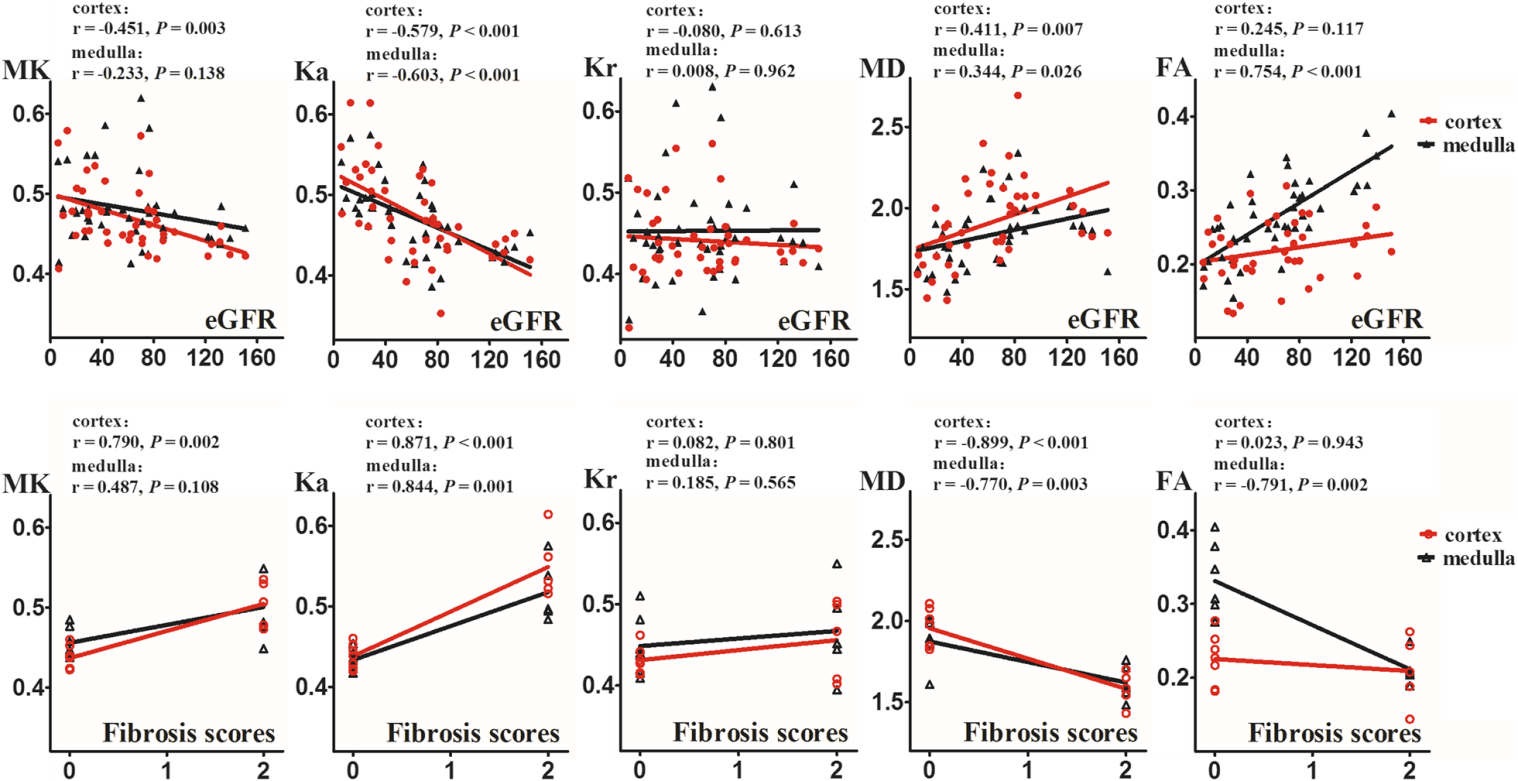
Scatter plots of correlations between eGFR (upper row), fibrosis (lower row) and DKI metrics of renal cortex and medulla. eGFR, estimated glomerular filtration rate; DKI, diffusion kurtosis imaging; MK, mean kurtosis; Ka, axial kurtosis; Kr, radial kurtosis; MD, mean diffusivity; FA, fractional anisotropy

**Fig. 5:**
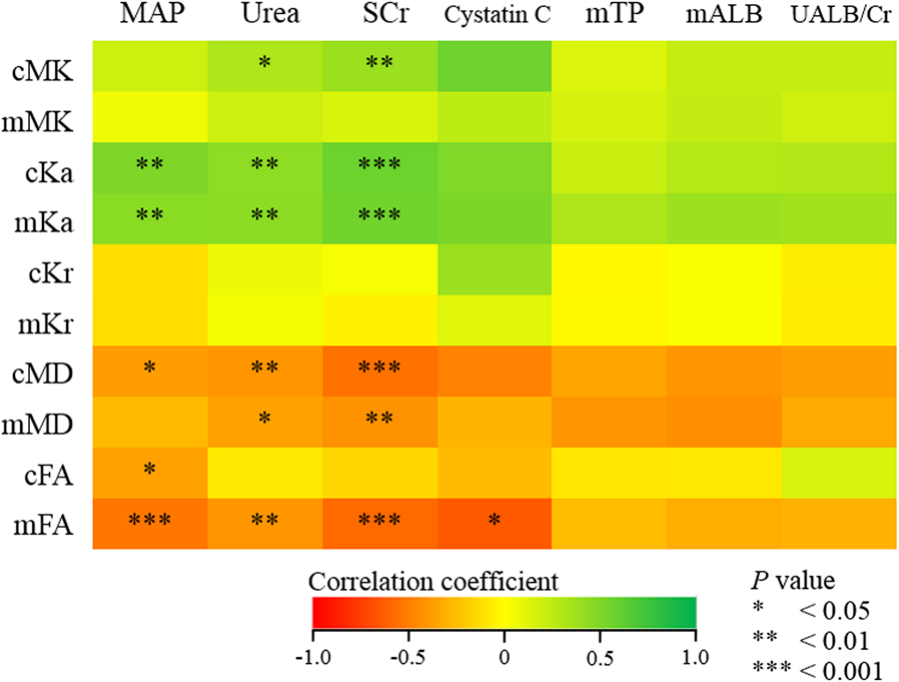
Heatmap of correlations between the clinicopathological characteristics of patients and DKI metrics of renal cortex (c) and medulla (m). DKI, diffusion kurtosis imaging; MAP, mean arterial blood pressure; SCr, serum creatinine; mTP, urinary total microprotein; mALB, urinary microalbumin; UALB/Cr, urinary microalbumin/creatinine

The correlations between DKI metrics and clinicopathological characteristics are shown in Figs. 4 and 5.

Cortical MK and parenchymal Ka were negatively correlated with eGFR (MK: *r* = − 0.451, *p* = 0.003; Ka: cortex: *r* = − 0.579, *p* < 0.001; medulla: *r* = − 0.603, *p* < 0.001), parenchymal MD and medullary FA were positively correlated with eGFR (MD: cortex: *r* = 0.411, *p* = 0.007; medulla: *r* = 0.344, *p* = 0.026; FA: *r* = 0.754, *p* < 0.001). However, there was no significant correlation between medullary MK, parenchymal Kr or cortical FA and eGFR (MK: *r* = − 0.233, *p* = 0.138; Kr: cortex: *r* = − 0.080, *p* = 0.613; medulla: *r* = 0.008, *p* = 0.962; FA: *r* = 0.245, *p* = 0.117).

Cortical MK and parenchymal Ka were positively correlated with fibrosis scores (MK: *r* = 0.790, *p* = 0.002; Ka: cortex: *r* = 0.871, *p* < 0.001; medulla: *r* = 0.844, *p* = 0.001), parenchymal MD and medullary FA were negatively correlated with fibrosis scores (MD: cortex: *r* = − 0.899, *p* < 0.001; medulla: *r* = − 0.770, *p* = 0.003; FA: *r* = − 0.791, *p* = 0.002). However, there was no significant correlation between medullary MK, parenchymal Kr or cortical FA and fibrosis scores (MK: *r* = 0.487, *p* = 0.108; Kr: cortex: *r* = 0.082, *p* = 0.801; medulla: *r* = 0.185, *p* = 0.565; FA: *r* = 0.023, *p* = 0.943).

Parenchyma Ka showed a positive correlation with MAP (cortex: *r* = 0.507, *p* = 0.001; medulla: *r* = 0.461, *p* = 0.003), cortical MD and parenchymal FA showed a negative correlated with MAP (MD: *r* = − 0.384, *p* = 0.014; FA: cortex: *r* = − 0.367, *p* = 0.020; medulla: *r* = − 0.533, *p* < 0.001). Cortical MK and parenchymal Ka were positively correlated with urea (MK: r = 0.322, *p* = 0.037; Ka: cortex: *r* = 0.446, *p* = 0.003; medulla: *r* = 0.453, *p* = 0.003), parenchymal MD and medullary FA were negatively correlated with urea (MD: cortex: *r* = − 0.408, *p* = 0.007; medulla: *r* = − 0.363, *p* = 0.018; FA: *r* = − 0.411, *p* = 0.007). Cortical MK and parenchymal Ka were positively correlated with Scr (MK: *r* = 0.398, *p* = 0.009; Ka: cortex: *r* = 0.581, *p* < 0.001; medulla: *r* = 0.557, *p* < 0.001), parenchymal MD and medullary FA were negatively correlated with Scr (MD: cortex: *r* = − 0.550, *p* < 0.001; medulla: *r* = − 0.427, *p* = 0.005; FA: *r* = − 0.582, *p* < 0.001). Medullary FA was negatively correlated with cystatin C (*r* = − 0.645, *p* = 0.044).

### Diagnostic performance of DKI for assessing the degrees of renal dysfunction

**Fig. 6:**
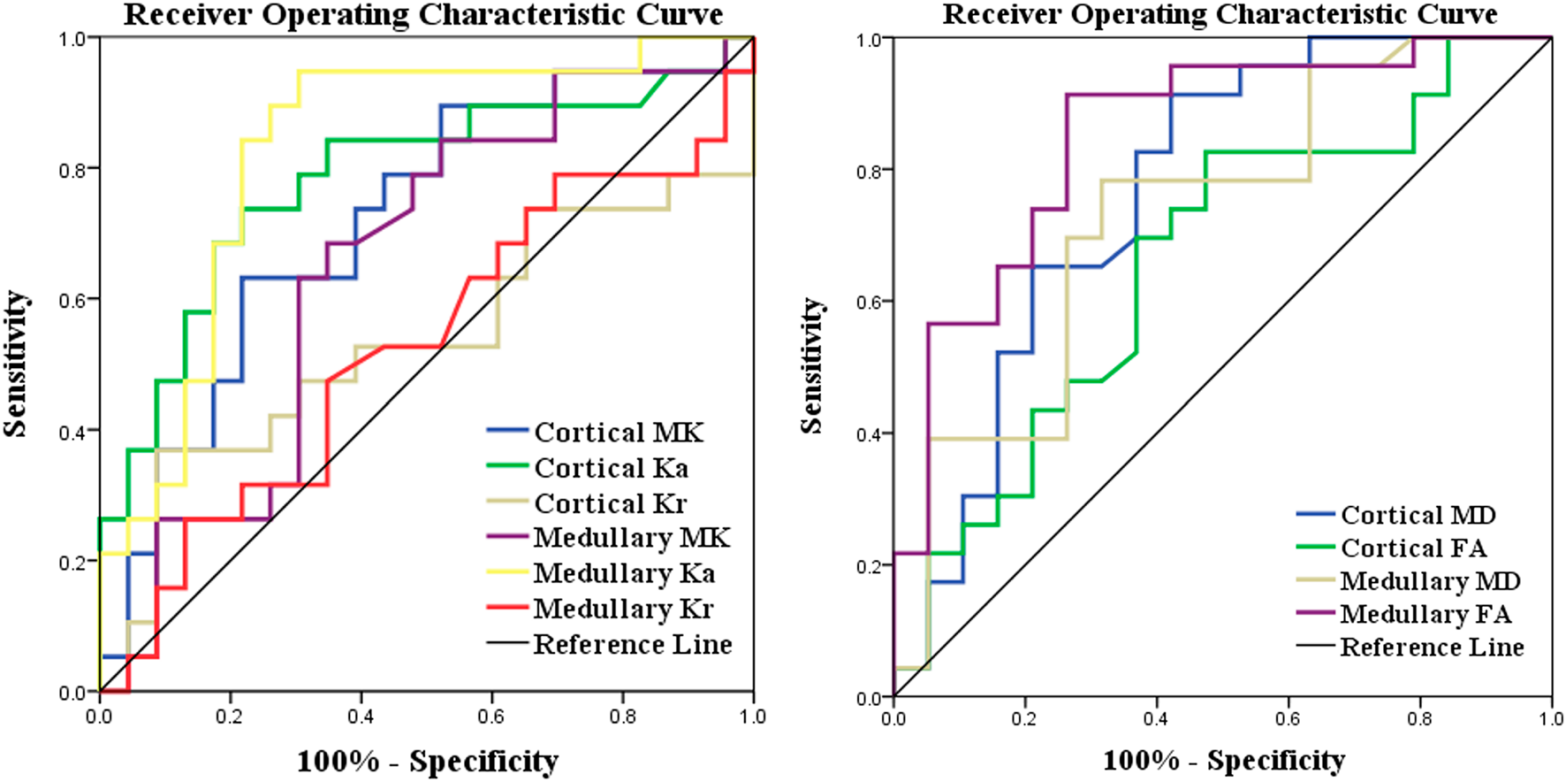
Receiver operating characteristic (ROC) curves of the DKI metrics in discriminating stable renal function from impaired renal function. ROC curves of medullary FA in assessing the degrees of renal dysfunction generated the highest area under the curve (AUC, 0. 955) among DKI metrics

**Fig. 7:**
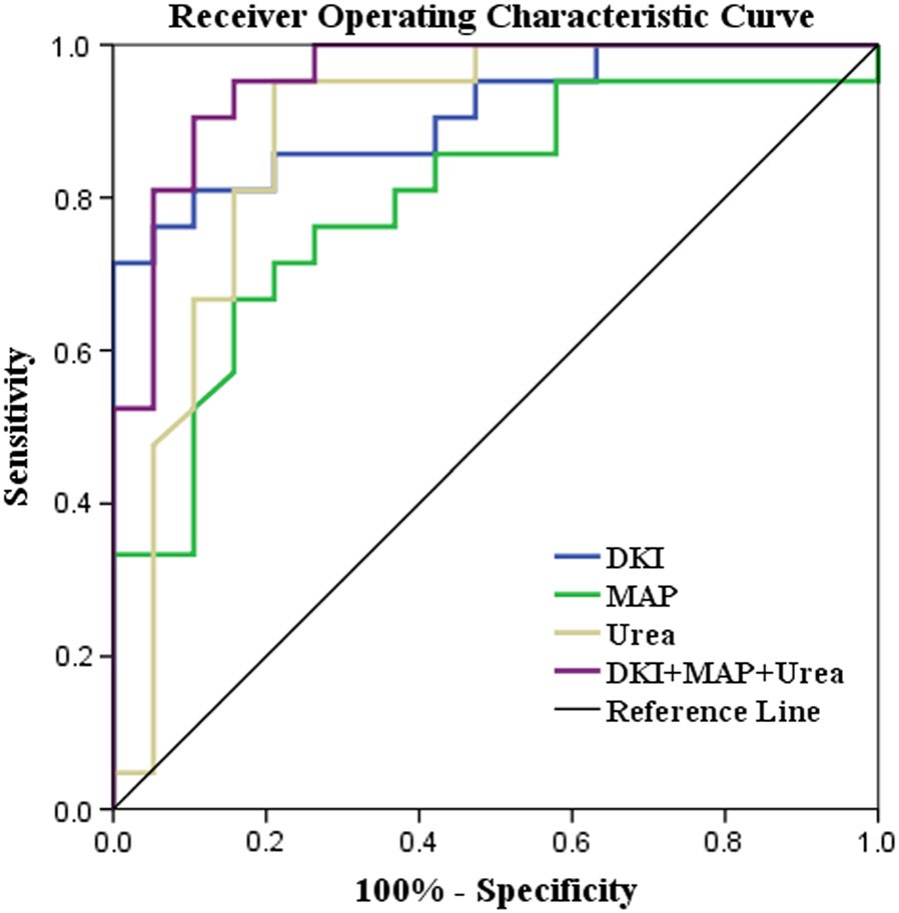
Receiver operating characteristic (ROC) curves of DKI and clinical indicators in discriminating stable renal function from impaired renal function. Combining DKI with MAP and urea can improve the diagnostic performance (AUC, 0. 972)

The diagnostic performance of DKI metrics and clinical indicators to discriminate stable renal function from impaired renal function is shown in Figs. 6, 7 and Tables 4, 5.

**Table 4:** Diagnostic performance of DKI metrics for assessing the degrees of renal dysfunction

	Youden index	Cutoff value	Sensitivity (%)	Specificity (%)	AUC	95% CI
**MK**						
Cortex	0.414	0.470	63.20	78.30	0.719	0.560–0.877
Medulla	0.336	0.476	68.40	65.20	0.652	0.483–0.821
**Ka**						
Cortex	0.519	0.469	73.70	78.30	0.785	0.638–0.931
Medulla	0.643	0.461	94.70	69.60	0.828	0.698–0.959
**Kr**						
Cortex	0.281	0.462	36.80	91.30	0.533	0.346–0.721
Medulla	0.133	0.487	26.30	87.00	0.526	0.346–0.707
**MD**						
Cortex	0.492	1.783	91.30	57.90	0.769	0.618–0.920
Medulla	0.467	1.779	78.30	68.40	0.731	0.575–0.887
**FA**						
Cortex	0.352	0.203	82.60	52.60	0.660	0.492–0.829
Medulla	0.650	0.249	91.30	73.70	0.851	0.732–0.971

*AUC* area under the receiver operating characteristic curve, *CI* confidence interval

**Table 5:** Diagnostic performance of DKI metrics and clinical indicators for assessing the degrees of renal dysfunction

	Youden index	Cutoff value	Sensitivity (%)	Specificity (%)	AUC	95% CI
DKI	0.714	–	71.40	100	0.910	0.820–0.999
MAP	0.509	101.83	66.70	84.20	0.796	0.655–0.937
Urea	0.742	8.85	95.20	78.90	0.883	0.767–0.999
DKI + MAP + Urea	0.799	–	90.50	89.50	0.955	0.895–1.000

*AUC* area under the receiver operating characteristic curve, *CI* confidence interval

Figure 6 and Table 4 showed the results of the ROC analyses of DKI metrics discriminate stable renal function from impaired renal function. Medullary FA achieved the highest area under the curve (AUC, 0. 851; Sensitivity, 91.3%; Specificity, 73.7%) among DKI metrics. Combining DKI with clinical indicators of MAP and urea showed the excellent diagnostic power for discrimination between stable renal function and impaired renal function (AUC, 0. 955; Sensitivity, 90.5%; Specificity, 89.5%) (Fig. 7 and Table 5).

## Discussion

To date, only few studies have explored the value of DKI technique for evaluating kidney disease in clinic [19–23]. Although our previous animal experiments have reported some promising findings, clinical patients have more complicated physiological conditions and pathological changes compared with animal models, the limited practicality of DKI may hinder its direct application in routine clinical practice [25]. In this preliminary study, we further explored the clinical application value of DKI for assessing progressive renal dysfunction and renal fibrosis.

In our study, MK, Kr, MD and FA between the renal cortex and medulla were significantly different, while no significant difference in Ka between cortex and medulla. Since the cortex is composed of renal corpuscles and tubules, and the medulla is composed of collecting ducts and part of tubules, it can expect that DKI metrics might differ between cortex and medulla [17]. However, as the disease progresses and renal function declined, the corticomedullary contrast was obscure, the difference in diffusion characteristics between cortex and medulla may decrease [27, 28]. Regarding the differences in DKI metrics between renal cortex and medulla, it was not surprising that the results of this study were inconsistent with those of our former animal experiment and other scholars’ researches [15–17, 22, 23, 25, 27, 29, 30]. There is no doubt that the different functional state between animal model and human body will inevitably lead to different results. DKI can better characterize the microstructural characteristics of tissues. It is worth noting that the existence of the abundant vasculature will complicate the measurement of the diffusion characteristics of the kidney. Although the influence of blood flow and vasculature of kidney on DKI have not been fully elucidated, studies have shown that corticomedullary difference of the kidney in DWI was sensitive to vascular flow. When interpreting DKI metrics, it is necessary to consider the potential impact of the renal vasculature on kurtosis value and diffusivity [15, 31]. In the future, we will combine with the biexponential intravoxel incoherent motion (IVIM) model to optimize the sequence and use advanced DKI protocol to further study the diffusion characteristics of kidney.

There were significant differences in cortical MK, parenchymal Ka, MD and medullary FA between stable and impaired eGFR patients as well as between mild and severe interstitial fibrosis patients, while there was no significant difference in Kr. Based on the results of our previous study, we found that with the deterioration of disease and the progressive decline of renal function, the glomerular capillary structure was gradually loss, tubular cell atrophy, tubules expand, inflammatory cells infiltration and extracellular matrix deposition [25]. These could lead to more complex microstructures than in normal kidneys, which greatly restricted the diffusion of water molecules and deviated from the Gaussian distribution. Therefore, the kurtosis values increases and the diffusivity decreases [16, 21]. Due to the dysfunction of radial transport of water molecules from vessels, tubules and collecting ducts to the renal pelvis in medulla, FA value decreased regardless of the cause of the damage to the renal pathology, physiology or microcirculation [30]. The results of this study were not exactly the same as those of previous clinical studies [21–23, 27, 29]. Because of kurtosis values will be affected by many factors. With the b value increases, the non-Gaussian diffusion properties become more prominent. Therefore, DWI acquired using high b values should make kurtosis estimates more robust. In addition, it is known that kurtosis value is also influenced by noise, magnetic field inhomogeneity and motion artifacts [15]. Even though the exact meaning of the kurtosis feature has not been fully interpreted, these discoveries still support the potential of DKI to disclose the changes of renal function and pathology.

Renal cortical MK, parenchymal Ka, MD and medullary FA were correlated with eGFR, interstitial fibrosis, urea and Scr, and renal parenchymal Ka, FA and cortical MD were also correlated with MAP, indicating that DKI could reflect renal function impaired and pathological changes. This was similar to the results of our animal experiments and other scholars’ studies [1, 20–23, 27, 29, 30]. Liu et al. showed that MK was not only well correlated with renal function on recruitment, eGFR slope and eGFR of the last visit in follow-up in CKD patients, but also significantly associated with the pathological score of fibrosis in IgA nephropathy [20, 21]. Mao et al. reported that cortical MK had the highest sensitivity for detecting alterations in renal function, MD is more sensitive than MK to detect renal fibrosis in CKD patients [22, 23]. Studies have also found that there was a negative correlation between FA and the stage of CKD, FA values decreases as the disease progresses [27, 30]. With the severity of renal damage, the progressive loss of glomerular capillary structures, the atrophy and stenosis of renal tubules, and the disappearance of glomerular cellular elements (replaced by expanding extracellular matrix and fibrous tissue) can lead to more complex and heterogeneous microstructure than in normal kidneys, cause an increase MK [20, 23, 24]. The glomerulosclerosis and tubulointerstitial fibrosis can lead to renal function impaired and blood flow and perfusion reduced, which making MD decreased [30]. The parenchymal FA value decreased with increased disease stage, possibly due to the involvement of renal microvessels, tubules and collecting ducts destroy the radial structure of kidneys [27]. Therefore, we can determine the progressive decline of renal function and fibrosis by DKI.

Among all the metrics of DKI, medullar FA has the highest diagnostic power for assessing the degree of renal dysfunction. Combining DKI with clinical indicators MAP and urea can further improve the diagnostic performance. This was similar to previous research [20, 21, 30]. The reason for the high diagnostic power of FA may be that the FA value mainly depends on the transport of water molecule in tubules, reflecting the direction characteristics of diffusion. Tubular atrophy eliminates part of directionality diffusion of water molecule along the cortex and medulla tubules. Moreover, glomerular lesion leads to the destruction of the structure and function of the filtration membrane, and macromolecular proteins and red blood cells can be filtered into tubules. Macromolecules and cellular debris can block the tubules and thereby affect the directed diffusion [30]. In addition, the medullary FA is determined in part by blood flow of kidney, and impaired renal parenchymal can lead to decreased blood flow and volume, as well as reduced of the medullary microcirculation [30, 32]. Renal insufficiency can be manifested as pathological damage to the glomeruli, tubules, interstitium or blood vessels and occur electrolyte and acid–base balance disorders due to filtration and reabsorption dysfunction, followed by endocrine dysfunction such as hypertension [33, 34]. Many studies have shown that clinical data and biopsy results have important roles in prediction of kidney disease progression and patient risk stratification, which has been widely recognized [1, 35]. Therefore, DKI combined with clinical indicators can get a preferable tool for comprehensive evaluation of renal function.

This study had some limitations. Firstly, the sample size was relatively small and devoid of follow-up. A larger sample size and complete clinical data may provide more precise diagnostic values and more information to predict the prognosis. Secondly, the abdominal DKI was influenced by respiratory motion from free-breathing imaging. But the kidney is retroperitoneal organ, motion artifact is relatively limited. In the future, we will optimize scheme using breath triggering or breath-hold imaging and perform image correction before analyzing. Thirdly, the quantitative value of DKI needs to be compared with other DWI models, such as monoexponential, triexponential and stretched exponential model, to further explore the potential advantages of DKI in assessing renal function and fibrosis. Fourthly, due to the low acceptance rate of renal puncture in clinic, biopsy was not performed in all patients in our study. We did not involve diagnostic performance of DKI for assessing the degrees of renal interstitial fibrosis or other histopathological characteristics. In response to this deficiency, our previous animal experiment has explored the correlation between DKI metrics and pathological results in UUO kidneys. In order to draw more definitive conclusions, it is necessary to fully compare the pathological results with DKI in a larger group of patients, and basic researches are also required to determine the precise mechanism of this process.

In conclusion, this preliminary study demonstrated that DKI have a certain value for noninvasive monitoring of renal function and interstitial fibrosis, timely identification of progressive renal dysfunction. More research is needed to verify the feasibility of this method for predicting the prognosis of kidney disease.

## Data Availability

Anonymized data are available upon request from the authors.

## Abbreviations

AUC: Area under the curve
BMI: Body mass index
DKI: Diffusion kurtosis imaging
DTI: Diffusion tensor imaging
eGFR: Estimated glomerular filtration rate
FA: Fractional anisotropy
Ka: Axial kurtosis
Kr: Radial kurtosis
mALB: Urinary microalbumin
MAP: Mean arterial blood pressure
MD: Mean diffusivity
MK: Mean kurtosis
mTP: Urinary total microprotein
ROC: Receiver operating characteristic
SCr: Serum creatinine
UALB/Cr: Urinary microalbumin/creatinine
